# Liver-Support Therapies in Critical Illness—A Comparative Analysis of Procedural Characteristics and Safety

**DOI:** 10.3390/jcm12144669

**Published:** 2023-07-13

**Authors:** Daniel Göth, Christoph F. Mahler, Florian Kälble, Claudius Speer, Louise Benning, Felix C. F. Schmitt, Maximilian Dietrich, Ellen Krautkrämer, Martin Zeier, Uta Merle, Christian Morath, Mascha O. Fiedler, Markus A. Weigand, Christian Nusshag

**Affiliations:** 1 Department of Nephrology, Heidelberg University Hospital, 69120 Heidelberg, Germany; daniel.goeth@med.uni-heidelberg.de (D.G.); christophfriedrich.mahler@med.uni-heidelberg.de (C.F.M.); florian.kaelble@med.uni-heidelberg.de (F.K.); claudius.speer@med.uni-heidelberg.de (C.S.); louise.benning@med.uni-heidelberg.de (L.B.); ellen.krautkraemer@med.uni-heidelberg.de (E.K.); martin.zeier@med.uni-heidelberg.de (M.Z.); christian.morath@med.uni-heidelberg.de (C.M.); 2 Department of Anesthesiology, Heidelberg University Hospital, 69120 Heidelberg, Germany; felix.schmitt@med.uni-heidelberg.de (F.C.F.S.); maximilian.dietrich@med.uni-heidelberg.de (M.D.); mascha.fiedler@med.uni-heidelberg.de (M.O.F.); markus.weigand@med.uni-heidelberg.de (M.A.W.); 3 Department of Gastroenterology, Heidelberg University Hospital, 69120 Heidelberg, Germany; uta.merle@med.uni-heidelberg.de

**Keywords:** liver-support therapy, acute liver dysfunction, acute liver failure, CytoSorb, MARS, therapeutic plasma exchange

## Abstract

Extracorporeal liver-support therapies remain controversial in critically ill patients, as most studies have failed to show an improvement in outcomes. However, heterogeneous timing and inclusion criteria, an insufficient number of treatments, and the lack of a situation-dependent selection of available liver-support modalities may have contributed to negative study results. We retrospectively investigated the procedural characteristics and safety of the three liver-support therapies CytoSorb, Molecular Adsorbent Recirculating System (MARS) and therapeutic plasma exchange (TPE). Whereas TPE had its strengths in a shorter treatment duration, in clearing larger molecules, affecting platelet numbers less, and improving systemic coagulation and hemodynamics, CytoSorb and MARS were associated with a superior reduction in particularly small protein-bound and water-soluble substances. The clearance magnitude was concentration-dependent for all three therapies, but additionally related to the molecular weight for CytoSorb and MARS therapy. Severe complications did not appear. In conclusion, a better characterization of disease-driving as well as beneficial molecules in critically ill patients with acute liver dysfunction is crucial to improve the use of liver-support therapy in critically ill patients. TPE may be beneficial in patients at high risk for bleeding complications and impaired liver synthesis and hemodynamics, while CytoSorb and MARS may be considered for patients in whom the elimination of smaller toxic compounds is a primary objective.

## 1. Introduction

As demographics change and multimorbidity increases, the numbers of critically ill patients requiring intensive care treatment are rising [1]. At the same time, mortality rates in these patients remain high, ranging from 11 to 12% regardless of underlying etiology, and even exceed 50% in patients requiring extracorporeal organ replacement therapies such as renal replacement therapy (RRT) [1,2,3,4]. The most common reasons for admission to the intensive care unit (ICU) include trauma, hemorrhagic or cardiogenic shock, and severe infections or sepsis, which all are not infrequently associated with consecutive (multi-) organ failure (MOV) [1,2]. As our understanding of the underlying pathophysiological processes and their drivers increases, new therapeutic strategies are being developed to positively influence disease progression by restoring body homeostasis. However, in situations where the underlying pathophysiological drivers are unknown or cannot be disrupted, organ replacement therapies play a crucial role in the treatment of critically ill patients. This holds especially true since organ failure or dysfunction itself negatively affects further disease progression by creating a toxic environment via the disruption of systemic metabolism, accumulation of toxic by-products, imbalance of body fluid compartments, and activation of proinflammatory cascades [5,6,7]. The establishment of RRT procedures to treat kidney failure represents a tremendous milestone in this context [8]. The same applies to critically ill patients with liver dysfunction or failure in whom various inflammatory mediators and toxins differ in lipophilicity, size and protein binding accumulate, resulting in a vicious cycle that further aggravates liver and multiorgan failure [7,9,10,11,12]. Acute liver failure (ALF) is a rare syndrome characterized by liver damage (elevated transaminases), impaired liver function (jaundice and prolongation of International Normalized Ratio [INR] ≥ 1.5) and encephalopathy in the absence of underlying chronic liver disease [12]. ALF is associated with poor outcomes and can be of primary (drug related, acute viral hepatitis, autoimmune, etc.) or secondary origin (sepsis, ischemic hepatitis, etc.) [12]. On the other hand, novel liver dysfunction without encephalopathy is more common in critical illness and can be mainly classified into two categories: hypoxic or ischemic hepatitis and cholestatic liver dysfunction [11]. Though a uniform definition is lacking, the latter is mostly referred to as acute liver dysfunction (ALD) and is likewise associated with significantly increased morbidity and mortality [13,14]. Especially in patients with cholestatic liver dysfunction, high bilirubin levels are a surrogate for the accumulation of toxic compounds such as bile acids and are associated with high mortality rates [14,15,16]. However, for ALF, and especially ALD, extracorporeal treatment options are less established, since available data are sparse or conflicting [10,17,18].

To date, the Molecular Adsorbent Recirculating System (MARS) is reported as the predominant extracorporeal liver-support system in the literature [7]. It is based on the concept of linking an albumin dialysis device to a regular renal replacement therapy system, allowing for the clearance of toxic water-soluble and protein-bound molecules [7,19]. MARS has been shown to improve bilirubin levels and hepatic encephalopathy in patients with ALF but has failed to demonstrate survival benefits in larger randomized controlled trials (RCTs) [7,20,21,22,23,24]. Therapeutic plasma exchange (TPE) is considered an alternative therapeutic option since it removes soluble and protein-bound toxins and, in contrast to MARS, counterbalances failing synthetic liver capacity by restoring liver proteins such as coagulation factors, albumin, complement factors and lipoproteins [9,17]. Recently, TPE has been shown to improve outcomes in patients with ALF by increasing transplant-free survival [17]. However, the overall evidence is still limited, and especially the role of TPE for other etiologies of liver dysfunction such as post-hepatectomy-associated liver failure or hypoxic liver failure in patients with ALD remains elusive [9,25]. Lastly, the extracorporeal blood purification device CytoSorb—initially developed for removing inflammatory mediators and cytokines in septic shock—is now suggested as a new treatment strategy in patients with liver dysfunction, since it adsorbs hydrophobic molecules of up to 55 kilo Daltons (kDa) in size and can be combined with standard RRT techniques [26,27,28,29,30]. Though CytoSorb has been shown to sufficiently remove bilirubin and bile acids via hemadsorption, the current data are limited to retrospective data, case series and in vitro data, and the expansion of its use to patients with ALF or ALD remains controversial [19,28,29,31,32,33].

As result, there is no clear recommendation as to when and in which patients the various liver-support modalities on the market are beneficial. Even for the most-studied therapy, MARS, it is controversial when and based on which parameters the therapy should be started and when it should be stopped. Thus, here we provide a direct comparison of the liver-support therapies CytoSorb, MARS and TPE, with a specific focus on therapeutic characteristics regarding the clearance of bilirubin and their effect on routine laboratory parameters, as well as on the individual risk profile of each therapy. A better understanding of clinical context-dependent advantages and disadvantages of these therapies is of relevance to improve the conceptual design of future studies. The latter are required to assess the true relevance of liver-support therapies in clinical routine.

## 2. Methods

### 2.1. Study Design

We performed a retrospective single-center analysis of critically ill patients treated with CytoSorb, MARS or TPE therapy between January 2016 and March 2022 at Heidelberg University Hospital, Germany. The retrospective observational study was approved by the local ethics committee with the purpose to improve the safety and treatment quality of critically ill patients with ALF or ALD at our center. For the present analyses, exclusively routine data were utilized. Extrahepatic cholestasis was excluded in all patients. As there were no study-related additional burdens for the patients, the study was approved by our ethics committee without informed consent according to German law and the exemption “§ 13 Abs. 1 LDSG BW. 1 LDSG BW”. Since both patients with ALF and ALD were included for the presented analyses, we calculated the Model for End-Stage Liver Disease (MELD) score and the Sequential Organ Failure Assessment (SOFA) score for a better comparison of disease severity prior to the first treatment. ALF was defined in accordance with the European Association for the Study of the Liver Guidelines [12]. CytoSorb filters (CytoSorbents Europe GmbH, Berlin, Germany) were combined with a standard hemodialysis machine (Genius^®^ therapy system, Fresenius Medical Care AG & Co. KGaA, Bad Homburg, Germany). MARS albumin dialysis (Baxter, Lund, Sweden) was attached to standard continuous renal replacement therapy (PRISMAFLEX, Baxter, Lund Sweden, continuous veno-venous hemodiafiltration [CVVHDF]) and used according to the manufacturer’s instructions. All three therapies were performed in accordance with the German Medical Devices Act (“Medizinproduktegesetz”) and are CE-marked (conformity for medical device) for the here-intended uses.

Unfractionated heparin or argatroban were used to provide adequate anticoagulation during CytoSorb and MARS treatments. Anticoagulant efficacy was monitored by measuring partial thromboplastin time (PTT). TPE was performed using a “Comtech” centrifuge (Fresenius Medical Care AG & Co. KGaA, Bad Homburg, Germany) and against fresh frozen plasma, exchanging a plasma volume of 50 mL/kg of body weight or a maximum of 4000 mL per treatment. Regional citrate anticoagulation was used as standard anticoagulation. 

### 2.2. Data Collection and Definitions

Patient characteristics, presented laboratory and clinical parameters, and complications associated with the individual treatment procedure were obtained from medical records. Hypotension was defined as a blood pressure drop of more than 20 mmHg during treatment. Hypocalcemia, -kalemia and -glycemia were defined as values below the standard values of the corresponding laboratory. Laboratory values measured were collected retrospectively before and after treatment from daily routine blood results. All laboratory parameters were measured in the accredited Central Laboratory of the Heidelberg University Hospital.

### 2.3. Statistical Analysis

Statistical analyses were performed using Graph Pad Prism 9 (GraphPad Software, La Jolla, CA, USA). Continuous variables are presented as median (interquartile range); categorical data are presented as absolute numbers (percentages). The Mann–Whitney U test was used for pairwise comparisons. Categorical variables were analyzed using the chi-square test. The Kruskal–Wallis test was used for multiple group comparisons. A *p*-value less than 0.05 was considered statistically significant. The correlation coefficient r was calculated with Spearman correlation. The R^2^ for data was computed using simple linear regression.

## 3. Results

### 3.1. Patient and Treatment Characteristics

Between January 2016 and March 2022, 21 patients were treated with the CytoSorb adsorber in a total of 60 treatment procedures. MARS therapy and TPE were performed in 14 patients with 61 treatments and in 18 patients with a total of 80 treatments, respectively (Table 1). Prior to the initiation of the respective liver-support therapy, all patients received guideline-based therapy for their individual underlying diseases, including RRT if appropriate. The indication for individual, extracorporeal liver-support therapy was set by the respective treating physician in a setting of non-resolving liver dysfunction despite the initiation of standard therapeutic measures. There were no predefined criteria for the respective liver-support therapy. However, in patients in whom the accumulation of toxic metabolites was considered a major concern (high bilirubin levels as surrogate)—and especially when an existing need for RRT was present—the preferred choice of therapy was CytoSorb or MARS treatment. On the other hand, patients with expected failing synthetic liver capacity, such as patients with early liver transplant failure, were more likely to receive TPE. The individual causes of liver dysfunction and patient characteristics are provided in Table 1. The predominant reasons in descending order were septic shock, cardiogenic shock, hemorrhagic shock, liver transplant failure, and liver failure after hemihepatectomy. With the exception of liver transplant failure, the etiology of liver dysfunction showed no significant differences and incidence of ALF was comparable across groups. There was no significant difference in age, gender, SOFA score and systolic blood pressure at baseline. MELD scores differed significantly at baseline, with the highest score achieved in patients treated with CytoSorb and MARS therapy. A tendency towards a higher vasopressor demand was observed in patients receiving MARS therapy. ICU mortality and length of ICU stay did not differ significantly across treatment groups. Further, comparing maximum liver parameters prior to the initiation of initial standard therapeutic measures, patients treated with MARS had higher transaminase levels than patients treated with CytoSorb and TPE, whereas alkaline phosphatase (AP), gamma-glutamyl transpeptidase (GGT) and International Normalized Ratio (INR) did not differ. However, indicating stable kinetics of liver parameters prior to the first extracorporeal treatment, the levels of liver biomarkers did not differ between the day of treatment and one day earlier for each liver-support therapy (Figure S1). Only total bilirubin increased significantly in MARS-treated patients, when comparing values between the day of treatment and the previous day. Lastly, with the exception of total bilirubin, routine laboratory biomarkers right before the first treatment did not differ between liver-support therapies (Table 1).

The procedural treatment characteristics are given in Table 2. Systemic anticoagulation with unfractioned heparin or argatroban was used for CytoSorb and MARS procedures, whereas TPE was performed using regional citrate anticoagulation. Moreover, 15% of MARS treatments were performed without additive anticoagulative therapy. Typical procedure-related differences were evident in the form of significantly higher blood flow rates for CytoSorb and MARS therapy compared to TPE procedures, and a shorter treatment duration for TPE therapy compared to CytoSorb and MARS therapy. Time from ICU admission to first treatment and the number of treatments per patient did not differ among the three groups studied. A mean plasma volume of 3.6 L was exchanged per TPE procedure.

### 3.2. Changes of Routine Laboratory Parameters in Response to Extracorporeal Liver Support Therapies

**Figure 1:**
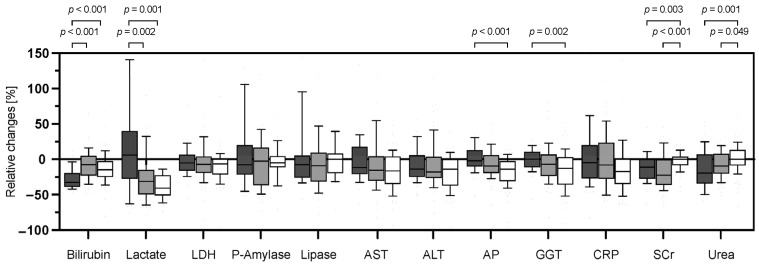
Procedure-dependent relative changes of laboratory parameters in response to CytoSorb, Molecular Adsorbent Recirculating System (MARS) and Therapeutic Plasma Exchange (TPE) therapy. Data are reported as box-and-whisker plots (interquartile range, 10% to 90% interval). Total bilirubin (Bilirubin); Lactate Dehydrogenase (LDH); Pancreas-Amylase (P-Amylase); Aspartate transaminase (AST); Alanine transaminase (ALT); Alkaline phosphatase (AP); Gamma-glutamyl Transpeptidase (GGT); C-reactive Protein (CRP); Serum creatinine (SCr).

**Figure 2:**
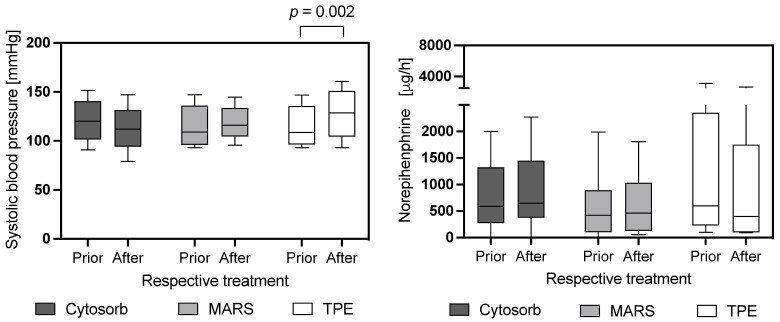
Procedure-dependent absolute changes of systolic blood pressure and vasopressor demand in response to CytoSorb, Molecular Adsorbent Recirculating System (MARS) and Therapeutic Plasma Exchange (TPE) therapy. Data are reported as box-and-whisker plots (interquartile range, 10% to 90% interval).

As shown in Figure 1 and Table S1, we found a procedure-associated reduction in most of the parameters measured. Across all three procedures, in descending order, the values of total bilirubin, lactate, AST, ALT and AP showed the most relevant reduction in association with the respective treatment compared to values before treatment. Specific procedure-dependent differences in laboratory parameters were seen for total bilirubin, lactate, AP and GGT. The decrease in total bilirubin was most effective using the CytoSorb system compared to MARS and TPE (Figure 1, Table 3 and Table 4, Tables S1 and S2). Lactate was most efficiently improved after TPE and MARS therapy, whereas AP and GGT showed the largest reduction in association with TPE. These findings apply to the evaluation of both absolute and relative changes in the analyzed parameters (Figure 1, Table S1). Serum creatinine (SCr) and urea reduction was more effective with CytoSorb and MARS than with TPE. The changes of laboratory parameters such as LDH, P-amylase, lipase, AST, ALT and CRP did not differ between the three treatment modalities, when all procedures were analyzed. The exclusive analysis of laboratory kinetics before and after the first treatment with CytoSorb, MARS or TPE of each patient is shown in Table S2. The absolute and relative reduction in laboratory parameters was more pronounced over the first TPE procedure, with a higher clearance of LDH, AST, ALT, AP, GGT and CRP in relation to CytoSorb and MARS therapy, as well as in comparison to the findings when all treatment procedures were analyzed. We further analyzed the correlation between the duration of treatment and the absolute reduction in individual laboratory parameters for the three liver-support modalities (Table S3). Here, we found no significant correlation between the treatment duration and associated changes in laboratory parameters. When comparing the procedure-related changes in systolic blood pressure and vasopressor requirements before and after each procedure, therapy with TPE resulted in a significant improvement in blood pressure compared with patients treated with CytoSorb and MARS, whereas the magnitude of vasopressor requirements did not change significantly with each therapy (Figure 2).

### 3.3. Concentration-Dependent Clearance of Different Liver-Support Modalities

To further elucidate a potential concentration-dependent effect on the reduction in individual laboratory parameters, correlation analyses of the concentration before and the absolute reduction after each treatment were performed. As illustrated in Table 3, we found a significant, concentration-dependent effect for almost all parameters studied, and for all three treatment modalities. For CytoSorb therapy, most relevant concentration-dependent effects (r < −0.5) were observed for the parameters bilirubin and ALT. MARS therapy showed the highest concentration-dependent effects for the reduction in total bilirubin, lactate, ALT, AP and GGT. Interestingly, with the exception of P-amylase and lipase, all other laboratory values studied showed a strong concentration-dependent clearance of the respective parameter under TPE therapy. Lastly, using total bilirubin as a surrogate parameter for the clearance of toxic protein-bound metabolites in ALD, we analyzed a range of matched bilirubin cut-offs and their effect on individual bilirubin clearance by CytoSorb, MARS and TPE therapy (Table 4). The efficacy of bilirubin removal was significantly reduced at levels below 15 mg/dL and 20 mg/dL for CytoSorb and MARS therapy, respectively, and almost no effect was observed at bilirubin levels below 10 mg/dL for either therapy. In contrast, the bilirubin clearance effect was largely maintained at levels above 10 mg/dL and was still effective in an attenuated form at levels below 10 mg/dL for TPE treatments.

**Table 3:** Correlation analysis of laboratory parameters before each treatment and their absolute reduction.

	CytoSorb				MARS				TPE			
Parameter	r	95% CI	*p* Value	R^2^	r	95% CI	*p* Value	R^2^	r	95% CI	*p* Value	R^2^
Total bilirubin	−0.79	−0.86–−0.70	0.001	0.63	−0.61	−0.75–−0.43	0.001	0.37	−0.55	−0.69–−0.37	0.001	0.44
Lactate	−0.23	−0.42–−0.01	0.034	0.13	−0.54	−0.71–−0.32	0.001	0.29	−0.70	−0.87–−0.40	0.001	0.47
LDH	0.15	−0.09–0.37	0.200	0.01	−0.42	−0.61–−0.20	0.001	0.18	−0.50	−0.65–−0.29	0.001	0.44
P-Amylase	−0.41	−0.61–−0.16	0.002	0.56	−0.35	−0.55–−0.11	0.006	0.12	−0.47	−0.75–−0.06	0.022	0.55
Lipase	−0.30	−0.54–−0.01	0.035	0.93	−0.49	−0.66–−0.27	0.001	0.24	−0.20	−0.57–+0.23	0.346	0.27
AST	−0.45	−0.61–−0.24	0.001	0.57	−0.45	−0.63–−0.23	0.001	0.21	−0.68	−0.78–−0.52	0.001	0.99
ALT	−0.51	−0.66–−0.32	0.001	0.71	−0.65	−0.77–−0.47	0.001	0.42	−0.77	−0.85–−0.65	0.001	0.95
AP	−0.35	−0.54–−0.14	0.001	0.08	−0.63	−0.76–−0.45	0.001	0.40	−0.71	−0.81–−0.58	0.001	0.94
GGT	−0.16	−0.37–+0.06	0.141	0.14	−0.54	−0.69–−0.33	0.001	0.29	−0.71	−0.81–−0.58	0.001	0.93
CRP	−0.34	−0.53–−0.12	0.002	0.03	−0.18	−0.42–+0.09	0.200	0.03	−0.67	−0.78–−0.51	0.001	0.44
SCr	−0.18	−0.45–+0.13	0.232	0.07	−0.57	−0.72–−0.37	0.001	0.23	−0.41	−0.58–−0.20	0.001	0.35
Urea	−0.13	−0.42–+0.17	0.378	0.02	−0.47	−0.65–−0.25	0.001	0.37	−0.31	0.51–−0.09	0.005	0.17

Molecular Adsorbent Recirculating System (MARS); Therapeutic Plasma Exchange (TPE); Lactate Dehydrogenase (LDH); Pancreas-Amylase (P-Amylase); Aspartate transaminase (AST); Alanine transaminase (ALT); Alkaline phosphatase (AP); Gamma-glutamyl Transpeptidase (GGT); Serum creatinine (SCr); C-reactive Protein (CRP).

**Table 4:** Cut-off values for total bilirubin and their effect on relative clerance by different liver-support modalities.

	CytoSorb			MARS			TPE		
Cut-Off Bilirubin	N	Median [%]	IQR [%]	N	Median [%]	IQR [%]	N	Median [%]	IQR [%]
>20 mg/dL	36	−33.8	−41.2–−24.3	5	−39.3	−48.1–−33.7	9	−28.4	−37.4–−23.7
>15–20 mg/dL	11	−32.0	−39.9–−23.0	16	−15.2	−28.3–−1.5	21	−17.2	−23.6–−2.1
>10–15 mg/dL	4	−14.4	−22.1–+5.5	33	−4.9	−12.7–+6.3	18	−20.3	−32.3–+8.2
≤10 mg/dL	6	+2.5	−9.9–+13.6	8	−0.9	−9.6–+9.8	29	−10.5	−20.9–−2.3

Molecular Adsorbent Recirculating System (MARS); Therapeutic Plasma Exchange (TPE). N indicates available datasets for each parameters before and after treatment.

### 3.4. Complications and Safety Analysis

As shown in Table 5, the safety profile of CytoSorb, MARS and TPE therapy was analyzed based on documented treatment-associated complications. In both the CytoSorb and MARS groups, clotting of the extracorporeal circuit occurred more frequently, whereas electrolyte disorders such as hypocalcemia and hypokalemia were present in the TPE group. The latter two disturbances were mild in nature and were compensated immediately during treatment procedures. Furthermore, we analyzed coagulation parameters before and after individual treatments. Therapy-associated platelet decline was most prominent for CytoSorb and MARS therapy compared to TPE procedures (Table 5). Furthermore, we noted an increase in INR levels under treatment with CytoSorb and MARS, whereas INR slightly improved in patients treated with TPE. Serious complications such as bleeding did not occur in any of the three groups studied. There was no documented case of transfusion reaction linked to TPE.

## 4. Discussion

The use of extracorporeal liver-support therapies in critically ill patients remains controversial, as most studies have failed to demonstrate improvements in hard outcomes [7,9,19,21,34]. Critically speaking, improvements in the laboratory results of different liver-support systems may stand in contrast to a meaningful improvement in the patient’s outcome and raises the question of whether we are treating ALF/ALD and its complications or rather laboratory numbers and the physician’s faith. Further, even if patients can be stabilized and show improvements in hepatic encephalopathy, cholestatic liver parameters and coagulation, it is unclear how long liver-support procedures should be continued, especially when liver transplantation is contraindicated and liver function fails to recover [7]. It is also worth mentioning that reliable evidence for MARS therapy comes mainly from patients with toxic and viral-induced ALF [7,22,23,35] or acute on chronic liver failure (AOCLF) based on alcohol or viral-related liver cirrhosis [7,20,21,24]. It is unclear whether or not these findings are transferable to other etiologies such as post-hepatectomy-associated or hypoxic liver dysfunction, or patients with ALD in general. This also applies to TPE and CytoSorb, as for TPE most studies are of a small sample size and concentrate on viral-induced liver diseases in patients with AOCLF, and data on CytoSorb mainly focus on the removal of bilirubin, ammonia and bile acids in patients with ALD [9,28,33,36,37,38].

On the other hand, there is evidence that heterogeneous timing, an insufficient number of treatments, and restrictive inclusion criteria, e.g., the exclusion of patients not eligible for liver transplantation, may have contributed to the negative study results [7,9]. In fact, two RCTs on TPE in ALF patients showed foremost a significant improvement in transplant-free survival, but this was not the case in patients receiving liver transplantation [17,39]. Consistent with this, a secondary analysis of the FLUMA trial found a significant improvement in survival in patients not receiving transplantation with ≥3 MARS sessions compared to those with <3 sessions [7,22]. Thus, RCTs comparing different liver-support modalities to standard medical treatment in patients with ALF or ALD are required to establish a better understanding of their true relevance in critically ill patients with liver failure.

We now provide, to our knowledge, the first comparison of the three liver-support systems CytoSorb, MARS and TPE regarding relevant treatment-related characteristics and the safety profile of each therapy. We clearly show that the three modalities differ in their ability and capacity to eliminate bilirubin as a surrogate of the accumulation of toxic metabolites such as bile acids, as well as water-soluble compounds such as urea, but also in their capacity to affect other routine laboratory parameters such as transaminases. Confirming in-vitro data comparing CytoSorb and MARS therapy, our data show that CytoSorb therapy is most effective for eliminating total bilirubin compared to MARS and TPE [31]. Though hyperbilirubinemia in adults has no direct toxic side effects (intact blood–brain barrier) and the magnitude of bile cast or cholemic nephropathy remains a matter of debate, bilirubin is a readily measurable surrogate for toxic bile acid accumulation in critically ill patients [40,41,42]. Given that bile acids have been shown to potentially cause hepatocyte damage and endothelial injury, and the fact that MARS and CytoSorb in particular are known to efficiently remove bile acids along with bilirubin, this highlights the relevance of our analyses and demonstrates that liver-supportive therapies may be appropriate in patients with non-resolving liver dysfunction [31,43]. On the other hand, MARS and TPE were superior in improving lactate levels—a crucial positive outcome surrogate in these patients—aligning with findings from previous studies [35,39]. Consistent with the results of TPE application in sepsis patients and in contrast to recent observations that CytoSorb leads to a reduction in vasopressors in patients with liver dysfunction, only TPE significantly improved hemodynamics in our analyses [33,44]. Though all three treatment modalities are capable of eliminating water-soluble and protein-bound molecules [7,9,27], assuming that the CytoSorb adsorber is linked to a RRT circuit, the clearance of SCr and urea was most effective using MARS or CytoSorb therapy in our cohort. The latter is most likely a consequence of the strong diffusive capacity of RRT techniques linked to the MARS device and CytoSorb adsorber at our center, and the longer treatment duration compared to TPE procedures. This may also be true for the removal of ammonia—a small, toxic, and water-soluble molecule that accumulates in patients with severe liver dysfunction—as it has been shown to be efficiently removed by high-flux membranes [45]. However, ammonia levels were not examined frequently enough in our patients, preventing a more detailed analysis in the present study. TPE, on the other hand, showed a superior clearance of larger molecules such as transaminases and especially LDH, AP, GGT and CRP—their molecular weight ranging from 45 kDa to around 140 kDa [46,47,48,49,50,51,52]—since TPE represents a rather nonselective removal technique [53]. The large, nonselective removal capacity of TPE was most evident after the first treatment, as in general approximately 66% and 85% of intravascular constituents were removed after the first and second TPE session, respectively [17,25]. Contrary to this, the adsorption capabilities of the CytoSorb system are limited to 55 kDa, and the MARS filter-membrane imposes a size selection threshold of around 50 kDa, explaining the finding observed in our study of an inferior reduction in particularly large molecules by both CytoSorb and MARS [27,54]. This may also suggest that the procedure-associated reduction in especially larger molecules such as ALT, AST, AP and GGT for CytoSorb and MARS therapy may imply that the decrease corresponds more to the resolution of liver injury or the improvement in liver function than to extracorporeal clearance. However, as the levels of these parameters were mostly stably elevated before the first treatment in our cohort, and a recent study made the same observation of a procedure-related reduction, a better understanding of their interaction with CytoSorb and MARS techniques is required [33].

Suggesting a concentration-dependent clearance efficacy, we further found a negative correlation between the extent of biomarker elevation prior to treatment and removal efficacy after treatment for all three liver-supportive therapies. While TPE showed strong concentration-dependent effects in almost all laboratory parameters studied, the extent of removal of larger molecules for CytoSorb and MARS therapy was less dependent on pretreatment concentration. When analyzing the matched cut-off-dependent clearance of bilirubin, CytoSorb and MARS therapy were best at removing bilirubin in patients with bilirubin levels beyond 20 mg/dL. However, the efficacy of bilirubin removal by CytoSorb and MARS decreased significantly at levels below 15 mg/dL and 20 mg/dL, respectively, and the removal efficacy was nearly lost at levels below 10 mg/dL. In contrast, TPE provided a lower but robust clearance capacity across all bilirubin levels higher than 10 mg/dL and was still effective in patients with bilirubin levels of 10 mg/dL and lower. This clearly shows that the clearance capacity depends on the biochemical properties and the liver-support system used but is also influenced by the present concentration of the target molecule in the individual patient. Interestingly, treatment duration had no effect on the extent of biomarker removal for the investigated liver-support therapies. This is most likely due to the fact that the mean treatment time of CytoSorb and MARS therapies in our cohort exceeded the binding capacity of the CytoSorb adsorbent and the charcoal and anion exchange columns in the MARS circuit, as recently shown in other studies [55,56]. For TPE procedures, treatment duration was largely comparable across patients, as it was mainly determined by the plasma volume exchanged. Also noteworthy in this context is the fact that, in contrast to MARS and CytoSorb, TPE does not allow for continuous solute and fluid control, highlighting the interrelated role of TPE together with RRT procedures in patients with severely impaired kidney and liver function.

Lastly, risk–benefit considerations are critical for prescribing potentially harmful extracorporeal therapies and highlight the need for a better understanding of individual risk profiles depending on the particular liver-support therapy and patient. In this context, our data show that all three liver-support therapies are safe, as no severe complications such as bleeding or transfusion reactions occurred, despite the need for system anticoagulation for MARS and CytoSorb therapy. Minor complications in the form of circuit clotting, mild hypokalemia and hypocalcemia, and hypotension occurred but were treated immediately during the course of treatment. This is consistent with the current evidence showing that clotting of the extracorporeal circulation is the most common complication of MARS, occurring in up to 23% of cases, while the risk of bleeding is no different from standard medical care [21,22,57]. Electrolyte disturbances associated with TPE for other indications are also common in the literature but are of minor concern as long as adequate therapy monitoring is ensured [58].

However, though severe therapy-associated bleeding complications appear rare, the three liver-support procedures nevertheless showed procedure-dependent differences with potential therapeutic implications for patients with impaired coagulation. Compared to TPE with 8%, a treatment-related reduction in platelet count was significantly more pronounced for CytoSorb and MARS therapy, with 26.5% and 18.4%, respectively. At the same time, consistent with previous studies, INR and hemodynamics improved under TPE since coagulation factors were substituted and blood pressure was raised by the administration of fresh plasma [9,17]. Therefore, TPE may be particularly beneficial in patients with low platelet counts, severely impaired liver synthesis and impaired hemodynamics, as seen in patients with shock and especially after hemihepatectomy with small-for-size syndrome. In line with this, a recent analysis suggested improved survival after hemihepatectomy for TPE compared to MARS therapy, although these results should be interpreted with caution given the small sample size and their retrospective nature [25]. Another potential advantage of TPE is its high efficacy despite shorter therapy intervals compared to CytoSorb and MARS procedures. This creates room for other important treatment aspects such as patient mobilization and allows for sequential treatment with adjunctive therapies such as RRT to remove water-soluble toxins. In contrast, methods such as MARS and CytoSorb may be of particular importance in patients where the removal of toxic protein-bound and water-soluble metabolites is a primary concern.

Some limitations of our study must be noted. The described treatment-related mortality rates should be interpreted with caution, given the retrospective nature of our analyses, the combined analysis of patients with ALF and ALD (heterogeneity of the population), the small sample size, and differing MELD scores, as well as a tendency for a higher vasopressor demand for MARS-treated patients at baseline. However, we want to point out that the main focus of our analyses was to identify the clinical context-dependent benefits and drawbacks of available liver-support modalities and their effect on routine laboratory parameters. In addition, as laboratory data were derived from daily routine blood results, factors other than individual liver-support therapies may have influenced the concentration of laboratory parameters. This holds especially true since the overall treatment-related improvement in hemodynamics may itself improve clinical and laboratory parameters. However, most laboratory parameters were comparable between the three treatment groups at baseline (Table 1). Further, after an initial improvement in liver parameters after the initiation of standard therapeutic measures, liver dysfunction persisted, and biomarker kinetics were mostly stable between the day of first treatment and the previous day (Figure S1). Lastly, the lack of clearly defined criteria for when to use one of the respective liver-support forms must be considered a limitation in the present retrospective analysis. Nevertheless, the optimal choice of therapy remains a challenge, as the right therapy and timing are not conclusively known based on current evidence.

## 5. Conclusions

Our data highlight several key aspects that may assist in improving the design of future studies addressing the relevance of liver-support therapies in critically ill patients with liver dysfunction. First, the clearance efficacy of specific molecules may depend not only on biochemical properties and the liver-support system used but also on the concentrations that are present in the individual patient at a certain time. Thus, a better characterization and differentiation of disease-driving, recovery-inhibiting and recovery-promoting molecules is indispensable to improve and personalize the use of extracorporeal liver-support techniques. Second, the available liver-support systems appear safe, especially if effective therapy monitoring and experience are provided. Third, TPE may be considered in patients with impaired liver synthesis, hemodynamics and coagulation or severe small-for-size syndrome, whereas CytoSorb or MARS procedures may have their advantages in removing smaller water-soluble or protein-bound toxic compounds. However, only comparative randomized trials of different liver-supportive therapies with standard medical treatment can properly assess their true significance for critically ill patients with severe liver dysfunction.

## Figures and Tables

**Table 1:** Baseline characteristics and associated outcomes.

	CytoSorb	MARS	TPE	*p* Value
Patients [*n*]	21	14	18	
Total treatments [*n*]	60	61	80	
Age [years]	54.0 (46.0–65.5)	58.5 (45.0–63.5)	58.5 (41.5–68.8)	0.955
Gender [male/female; %]	71.0/29.0	64.3/35.7	61.1/38.9	0.313
SOFA Score prior to first treatment	15.0 (9.0–16.5)	14.0 (11.0–17.3)	11.0 (5.0–17.0)	0.368
MELD Score prior to first treatment	34.0 (33.0–36.5)	34.0 (29.0–36.0)	29.0 (22.8–33.5)	0.005
Vasopressors prior to first treatment [*n*, (%)]	12 (57)	12 (86)	10 (56)	0.145
Systolic blood pressure prior to treatment [mmHg]	120.0 (101.5–140.5)	117.0 (104.8–134.3)	108.5 (96.3–135.5)	0.227
Acute liver failure [*n*, (%)]	9 (42.9%)	6 (42.9%)	8 (44.4%)	0.994
Liver parameter maximum				
Total bilirubin [mg/dL]	25.5 (19.8–36.6)	17.8 (13.1–21.3)	19.1 (10.9–23.7)	0.011
AST [U/L]	727.0 (184.5–4729.0)	5228.0 (1492.0–10,916.0)	615.0 (241.0–5548.0)	0.032
ALT [U/L]	269.0 (91.0–1424.0)	3288.0 (467.0–4715.0)	430.0 (204.3–3413.0)	0.038
AP [U/L]	257.5 (168.0–408.3)	333.5 (245.3–658.0)	297.5 (156.0–502.5)	0.408
GGT [U/L]	153.0 (78.5–355.5)	150.0 (102.0–670.3)	132.0 (82.0–373.5)	0.816
INR	1.96 (1.48–3.38)	2.97 (2.06–3.57)	2.08 (1.45–2.82)	0.094
Etiology of liver dysfunction				
Septic shock [*n*, (%)]	10 (48)	7 (50)	8 (44)	0.951
Pulmonary focus	2 (20)	2 (29)	2 (25)	0.918
Abdominal focus	5 (50)	5 (71)	6 (75)	0.487
Urogenital focus	1 (10)	0 (0)	0 (0)	0.458
Unknown focus	2 (20)	0 (0)	0 (0)	0.196
Hypoxic liver injury [*n*, (%)]	6 (29)	3 (21)	2 (11)	0.608
Cardiogenic shock	3 (50)	1 (33)	0 (0)	0.441
Hemorrhagic shock	3 (50)	0 (0)	1 (50)	0.308
Vascular occlusion	0 (0)	2 (66)	1 (50)	0.087
Liver transplant failure [*n*, (%)]	0 (0)	0 (0)	4 (22)	0.015
Hemihepatectomy [*n*, (%)]	2 (10)	4 (29)	1 (6)	0.132
Toxic [*n*, (%)]	1 (5)	0 (0)	0 (0)	0.460
Autoimmune hepatitis [*n*, (%)]	0 (0)	0 (0)	2 (11)	0.133
Cryptogenic [*n*, (%)]	2 (1)	0 (0)	1 (6)	0.490
Laboratory parameters prior to first treatment				
Total bilirubin [mg/dL]	22.2 (18.3–29.3)	14.2 (8.6–17.9)	15.3 (10.8–20.0)	0.002
Lactate [mg/dL]	19.0 (12.6–31.9)	32.1 (23.6–48.7)	28.4 (23.9–39.0)	0.072
LDH [U/L]	424.5 (272.3–739.5)	447.0 (269.0–1005.0)	407.0 (308.0–666.5)	0.947
P-Amylase [U/L]	20.0 (12.0–75.0)	38.5 (24.8–149.8)	35.0 (12.5–96.0)	0.316
Lipase [U/L]	42.5 (20.3–77.0)	82.5 (25.5–200.3)	35.0 (26.5–140.3)	0.470
AST [U/L]	113.5 (75.8–506.3)	231.5 (105.0–758.3)	235.0 (99.0–1113.0)	0.406
ALT [U/L]	120.0 (69.0–277.0)	163.0 (53.5–709.8)	202.0 (74.5–1056.0)	0.551
AP [U/L]	188.0 (135.0–292.0)	193.5 (89.3–356.5)	226.0 (130.5–370.5)	0.816
GGT [U/L]	93.5 (54.0–208.5)	127.5 (62.8–451.8)	83.0 (49.0–234.5)	0.660
CRP [mg/L]	69.6 (42.7–123.3)	52.4 (40.0–94.6)	29.9 (9.3–127.0)	0.464
SCr [mg/dL]	1.4 (0.7–2.8)	1.6 (1.1–2.3)	1.0 (0.6–1.7)	0.101
Urea [mg/dL]	63.0 (44.0–175.5)	69.0 (50.3–90.8)	51.5 (25.8–80.0)	0.169
Platelets [/nL]	52.5 (39.5–115.5)	62.5 (47.5–137.8)	74.0 (34.5–135.5)	0.693
Albumin [g/L]	28.4 (24.2–35.1)	30.6 (28.2–32.5)	29.6 (24.8–31.4)	0.498
INR	1.3 (1.2–1.7)	1.6 (1.3–1.8)	1.4 (1.2–2.2)	0.359
PTT [s]	32.8 (29.8–44.3)	34.4 (27.6–43.0)	30.8 (26.8–39.5)	0.596
*Associated outcomes*				
Length of ICU stay [days]	21.0 (13.0–49.0)	25.0 (12.3–34.5)	21.0 (12.0–32.0)	0.840
Mortality in ICU [*n*, (%)]	16 (76.2)	9 (64.3)	8 (44.4)	0.128
RRT requirement [*n*, (%)]	15 (71)	12 (86)	8 (44)	0.040

Molecular Adsorbent Recirculating System (MARS); Therapeutic Plasma Exchange (TPE); Renal Replacement Therapy (RRT); Sequential Organ Failure Assessment (SOFA); model for end-stage liver disease (MELD); Intensive Care Unit (ICU); partial thromboplastin time (PTT); International Normalized Ratio (INR), Lactate Dehydrogenase (LDH); Pancreas-Amylase (P-Amylase); Aspartate transaminase (AST); Alanine transaminase (ALT); Alkaline phosphatase (AP); Gamma-glutamyl Transpeptidase (GGT); C-reactive Protein (CRP), Serum creatinine (SCr).

**Table 2:** Procedure-dependent treatment characteristics.

Treatment Parameter	CytoSorb	MARS	TPE	*p* Value
Duration of treatment [hours]	12.0 (8.4–17.0)	14.5 (8.0–19.0)	2.2 (1.8–2.3)	<0.001
Blood pump flow rate [mL/min]	200.0 (160.0–200.0)	150.0 (150.0–150.0)	47.0 (43.0–50.0)	<0.001
Albumin flow rate [mL/min]	-	150.0 (150.0–150.0)	-	
Centrifuge plasma flow rate [mL/min]	-	-	31.0 (30.0–32.0)	
Exchanged plasma volume [L]	-	-	3.6 (3.6–3.9)	
Anticoagulation with heparin [*n*]	60 (100%)	25 (41%)	0 (0%)	<0.001
Anticoagulation with argatroban [*n*]	0 (0%)	27 (44%)	0 (0%)	<0.001
Anticoagulation with citrate [*n*]	0 (0%)	0 (0%)	80 (100%)	<0.001
No anticoagulation [*n*]	0 (0%)	9 (15%)	0 (0%)	<0.001
PTT during treatment	64.0 (37.2–113.2)	47.3 (39.8–54.7)	27.7 (24.4–30.1)	<0.001
INR during treatment	1.3 (1.2–1.8)	1.8 (1.4–2.1)	1.3 (1.2–1.4)	<0.001
Treatments per patient [*n*]	2.0 (1.0–4.5)	2.5 (1.0–5.0)	3.0 (1.0–6.5)	0.353
Combination with RRT [*n*]	43 (71%)	61 (100%)	0 (0%)	<0.001
Time from ICU admission to first treatment [days]	12.0 (5.5–37.0)	6.5 (2.0–16.3)	7.0 (2.0–13.0)	0.132

Molecular Adsorbent Recirculating System (MARS); Therapeutic Plasma Exchange (TPE); partial thromboplastin time (PTT); International Normalized Ratio (INR); Intensive Care Unit (ICU); Renal replacement therapy (RRT).

**Table 5:** Therapy-associated complications and coagulation parameters.

Parameters	CytoSorb	MARS	TPE	*p* Value
Clotting [*n*]	13 (21%)	16 (26%)	2 (3%)	<0.001
Hypotension [*n*]	2 (1.8%)	2 (3%)	1 (1%)	0.657
Hypocalcemia [*n*]	0 (0%)	0 (0%)	10 (13%)	0.001
Hypokalemia [*n*]	0 (0%)	0 (0%)	16 (20%)	0.001
Bleeding [*n*]	0 (0%)	0 (0%)	0 (0%)	
Transfusion reaction	-	-	0 (0%)	
Platelet reduction				
Absolute changes [/nL]	−17.0 (−33.0–−7.0)	−10.5 (31.0–+2.5)	−7.0 (−19.0–+1.0)	0.071
Relative changes [%]	−26.5 (−41.2–−6.8)	−18.4 (−35.5–+7.6)	−8.0 (−25.0–+1.6)	0.024
INR changes				
Absolute changes	+0.1 (0–+0.5)	+0.03 (−0.3–+0.2)	−0.02 (−0.1–0)	0.241
Relative changes [%]	+5.5 (+0.7–+31.6)	+9.1 (−7.5–+39.6)	−1.6 (−5.0–+1.6)	<0.001

Molecular Adsorbent Recirculating System (MARS); Therapeutic Plasma Exchange (TPE); International Normalized Ratio (INR).

## Data Availability

The data presented in this study are not publicly available due to ethical restrictions.

## Supplementary Materials

The following supporting information can be downloaded at: https://www.mdpi.com/article/10.3390/jcm12144669/s1, Table S1: Relative and absolute changes of laboratory parameters in response to CytoSorb, MARS and TPE treatments (all treatments). Table S2: Relative and absolute changes after first treatment of CytoSorb, MARS and TPE therapy; Table S3: Correlation analysis of absolute reduction of parameters studied and duration of treatment; Figure S1: Course of routine liver parameters within two days before first liver support therapy.
