# Supplementary material for: Liver-Support Therapies in Critical Illness—A Comparative Analysis of Procedural Characteristics and Safety
Source: J Clin Med. 2023 Jul 13;12(14):4669. doi: 10.3390/jcm12144669 (PMC10380554; doi:10.3390/jcm12144669)
Supplement: Supplementary file 1 [file jcm-12-04669-s001.zip › jcm-2377419-supplementary.pdf]

Journal of
Clinical Medicine

MDPI

*Supplementary Materials*

# Liver-Support Therapies in Critical Illness—A Comparative Analysis of Procedural Characteristics and Safety

## Supplemental Tables

**Table S1.** Relative and absolute changes of laboratory parameters in response to CytoSorb, MARS and TPE treatments (all treatments).

| | CytoSorb | | | MARS | | | TPE | | | |
|---|---|---|---|---|---|---|---|---|---|---|
| **Relative changes [%]** | ***n*** | **Median** | **IQR** | ***n*** | **Median** | **IQR** | ***n*** | **Median** | **IQR** | ***p* Value** |
| Total bilirubin | 51 | -32.0 | -38.1 – -19.6 | 63 | -7.5 | -4.7 – +23.0 | 77 | -17.9 | -26.2 – -4.3 | <0.001 |
| Lactate | 44 | -5.7 | -24.6 – +38.9 | 53 | -30.9 | +15.0 – +48.0 | 22 | -40.6 | -50.2 – -25.0 | <0.001 |
| LDH | 46 | -5.5 | -15.8 – +6.2 | 63 | -7.0 | -3.7 – +18.4 | 76 | -6.5 | -19.1 – +1.2 | 0.415 |
| P-Amylase | 26 | -7.4 | -21.0 – +15.6 | 61 | -2.5 | -14.3 – +36.4 | 23 | -5.0 | -10.9 – +2.3 | 0.778 |
| Lipase | 26 | -6.5 | -22.9 – +3.2 | 61 | -9.1 | -9.1 – +31.0 | 24 | -1.6 | -15.9 – +8.3 | 0.540 |
| AST | 49 | -13.3 | -20.4 – +15.8 | 62 | -15.3 | -3.0 – +30.0 | 76 | -17.4 | -36.9 – +4.0 | 0.120 |
| ALT | 48 | -14.0 | -23.8 – +5.5 | 62 | -18.0 | -2.7 – +26.0 | 76 | -16.1 | -37.0 – -1.6 | 0.215 |
| AP | 48 | -3.1 | -9.6 – +11.8 | 62 | -9.3 | -5.7 – +19.0 | 76 | -13.1 | -29.0 – -2.6 | <0.001 |
| GGT | 50 | -5.0 | -10.5 – +10.4 | 62 | -6.9 | -6.3 – +22.0 | 76 | -12.7 | -34.6 – +3.2 | 0.002 |
| CRP | 44 | -4.6 | -26.7 – +18.7 | 55 | -12.4 | -22.8 – +26.2 | 67 | -16.7 | -34.0 – 0 | 0.117 |
| SCr | 46 | -11.0 | .27.3 – +0.3 | 62 | -22.4 | -36.0 – 0.0 | 77 | 0.0 | -8.1 – +3.7 | <0.001 |
| Urea | 46 | -19.1 | -34.2 – +7.0 | 62 | -9.6 | -20.1 – +7.9 | 77 | 0.0 | -9.6 – +12.9 | 0.001 |
| **Absolute changes** | ***n*** | **Median** | **IQR** | ***n*** | **Median** | **IQR** | ***n*** | **Median** | **IQR** | ***p* Value** |
| Total bilirubin [mg/dl] | 51 | -6.9 | -9.5 – -4.2 | 63 | -0.7 | -3.2 – +0.5 | 77 | -1.6 | -0.3 – -3.9 | <0.001 |
| Lactate [mmol/l] | 44 | +0.6 | -5.0 – +5.0 | 53 | -7.3 | -12.0 – +2.3 | 22 | -12.7 | -8.2 – -18.1 | <0.001 |
| LDH [U/l] | 46 | -24.0 | -65.5 – +15.5 | 63 | -18.0 | -74.0 – +13.0 | 76 | -22.0 | -84.0 – +1.0 | 0.655 |
| P-Amylase [U/l] | 26 | -1.0 | -7.5 – +1.0 | 61 | -1.0 | -29.0 – +6.0 | 23 | -1.0 | -5.5 – +0.5 | 0.934 |
| Lipase [mg/dl] | 26 | -2.0 | -9.5 – +1.5 | 61 | -2.0 | -26.0 – +5.0 | 24 | -0.5 | -5.5 – +1.8 | 0.660 |
| AST [U/l] | 49 | -9.0 | -21.8 – +14.8 | 62 | -19.0 | -107.0 – +3.0 | 76 | -13.0 | -92.0 – +3.0 | 0.117 |
| ALT [U/l] | 48 | -10.0 | -29.0 – +4.0 | 62 | -14.0 | -44.0 – +3.5 | 76 | -10.0 | -58.0 – -1.0 | 0.5133 |
| AP [U/l] | 48 | -7.0 | -40.0 – +35.0 | 62 | -21.0 | -56.0 – +6.8 | 76 | -18.0 | -43.0 – -3.0 | 0.047 |
| GGT [U/l] | 50 | 1.0 | -14.5 – +11.5 | 62 | -9.5 | -54.0 – +4.0 | 76 | -9.0 | -27.0 – +1.0 | 0.031 |
| CRP [mg/l] | 44 | -2.2 | -9.2 – +4.2 | 55 | -4.3 | -22.0 – +8.9 | 67 | -1.0 | -5.9 – 0 | 0.934 |
| SCr [mg/dl] | 46 | -0.1 | -0.3 – 0.0 | 62 | -0.2 | -0.5 – 0.0 | 77 | 0.0 | -0.1 – 0.0 | <0.001 |
| Urea [mg/dl] | 46 | -6.5 | -23.3 – +3.3 | 62 | -8.0 | -20.5 – +5.3 | 77 | 0.0 | -5.0 – +4.5 | 0.007 |

Molecular Adsorbent Recirculating System (MARS); Therapeutic Plasma Exchange (TPE); Lactate Dehydrogenase (LDH); Pancreas-Amylase (P-Amylase); Aspartate transaminase (AST); Alanine transaminase (ALT); Alkaline phosphatase (AP); Gamma-glutamyl Transpeptidase (GGT); C-reactive Protein (CRP); Interquartile range (IQR); Serum creatinine (SCr). *n* indicates available datasets for each parameter before and after treatment.

**Table S2.** Relative and absolute changes after first treatment of CytoSorb, MARS and TPE therapy.

| | CytoSorb | | | MARS | | | TPE | | | |
|---|---|---|---|---|---|---|---|---|---|---|
| **Relative changes [%]** | ***n*** | **Median** | **IQR** | ***n*** | **Median** | **IQR** | ***n*** | **Median** | **IQR** | ***p* Value** |
| Total bilirubin | 19 | -33.2 | -39.5 – -19.4 | 14 | -8.9 | -26.3 – +5.7 | 17 | -24.6 | -34.2 – -5.0 | 0.140 |
| Lactate | 19 | -14.8 | -42.8 – +36.0 | 10 | -42.5 | -64.0 – -0.35 | 9 | -39.8 | -45.6 – -23.3 | 0.280 |
| LDH | 19 | -8.6 | -17.3 – +6.4 | 14 | -5.2 | -11.4 – +34.8 | 17 | -26.6 | -40.0 – -8.6 | 0.002 |
| P-Amylase | 13 | -7.7 | -21.0 – +15.6 | 13 | +0.0 | -30.0 – +24.6 | 9 | -9.5 | -26.8 – -2.5 | 0.406 |
| Lipase | 13 | -16.2 | -27.8 – -0.9 | 13 | +0.0 | -7.9 – +12.7 | 10 | -17.1 | -28.6 – -7.7 | 0.033 |
| AST | 19 | -16.9 | -24.2 – +17.5 | 14 | -14.2 | -33.0 – +51.3 | 17 | -44.8 | -60.9 – -29.4 | <0.001 |
| ALT | 18 | -14.5 | -26.1 – +9.7 | 14 | -16.9 | -27.0 – +9.6 | 17 | -50.9 | -59.4 – -37.1 | <0.001 |
| AP | 16 | +3.7 | -9.0 – +15.0 | 14 | -7.5 | -21.1 – +15.2 | 17 | -37.1 | -49.3 – -24.3 | <0.001 |

| | | | | | | | | | | |
|---|---|---|---|---|---|---|---|---|---|---|
| GGT | 19 | -2.2 | -11.8 – +11.1 | 13 | -11.8 | -27.5 – +9.6 | 17 | -42.3 | -58.0 – -31.8 | <0.001 |
| CRP | 19 | -5.6 | -29.9 – +7.9 | 11 | +3.0 | -26.9 – + 50.8 | 12 | -34.4 | -52.0 – -3.8 | 0.037 |
| SCr | 20 | -9.2 | -26.7 – +0.8 | 13 | -29.2 | -34.9 – -20.5 | 17 | -12.2 | -20.0 – +5.2 | 0.011 |
| Urea | 20 | -19.8 | -31.9 – +6.7 | 13 | -3.7 | -27.8 – +8.7 | 17 | -3.8 | -15.9 – +10.2 | 0.558 |
| **Absolute changes** | ***n*** | **Median** | **IQR** | ***n*** | **Median** | **IQR** | ***n*** | **Median** | **IQR** | ***p* Value** |
| Total bilirubin [mg/dl] | 19 | -6.0 | -8.8 – -3.4 | 14 | -0.9 | -4.7 – +0.3 | 17 | -3.7 | -7.2 – -0.4 | 0.017 |
| Lactate [mmol/l] | 19 | -2.8 | -11.5 – +5.7 | 10 | -8.1 | -30.3 – +0.5 | 9 | -12.7 | -15.1 – -6.4 | 0.176 |
| LDH [U/l] | 19 | -29.0 | -116.0 – +18.0 | 14 | -13.0 | -62.5 – +140.5 | 17 | -88.0 | -191.0 – -54.5 | 0.011 |
| P-Amylase [U/l] | 13 | -1.0 | -10.0 – +1.0 | 13 | +0.0 | -12.5 – +37.0 | 9 | -5.0 | -20.5 – -0.5 | 0.244 |
| Lipase [mg/dl] | 13 | -3.0 | -15.0 – +30.0 | 13 | +0.0 | -4.0 – +30.0 | 10 | -6.0 | -15.0 – -1.0 | 0.086 |
| AST [U/l] | 19 | -13.0 | -42.0 – +19.0 | 14 | -20.0 | -144.8 – +56.0 | 17 | -104.0 | -640.0 – -36.0 | 0.004 |
| ALT [U/l] | 18 | -13,0 | -52.3 – +6.5 | 14 | -12.0 | -133.8 – +6.8 | 17 | -120.0 | -604.5 – -22.0 | 0.010 |
| AP [U/l] | 16 | +0.5 | -23.5 – +24.8 | 14 | -21.5 | -57.3 – +12.0 | 17 | -83.0 | -161.0 – -32.0 | 0.001 |
| GGT [U/l] | 19 | -2.0 | -13.0 – +9.0 | 13 | -32.5 | -104.8 – +1.8 | 17 | -43.0 | -167.0 – -15.5 | 0.001 |
| CRP [mg/l] | 19 | -2.4 | -27.8 – +7.5 | 11 | +6.1 | -22.0 – +24.5 | 12 | -4.4 | -26.5 – +1.4 | 0.178 |
| SCr [mg/dl] | 20 | -0.1 | -0.3 – 0.0 | 13 | -0.5 | -0.8 – -0.3 | 17 | -0.2 | -0.3 – 0.0 | 0.011 |
| Urea [mg/dl] | 20 | -6.0 | -23.8 – +7.8 | 13 | -3.0 | -23.0 – +6.0 | 17 | -2.0 | -17.0 – +2.5 | 0.908 |

Molecular Adsorbent Recirculating System (MARS); Therapeutic Plasma Exchange (TPE); Lactate Dehydrogenase (LDH); Pancreas-Amylase (P-Amylase); Aspartate transaminase (AST); Alanine transaminase (ALT); Alkaline phosphatase (AP); Gamma-glutamyl Transpeptidase (GGT); C-reactive Protein (CRP); Interquartile range (IQR); Serum Creatinine (SCr). *n* indicated available datasets for each parameters before and after treatment.

**Table S3.** Correlation analysis of absolute reduction of parameters studied and duration of treatment.

| | **CytoSorb** | | | | **MARS** | | | | **TPE** | | | |
|---|---|---|---|---|---|---|---|---|---|---|---|---|
| **Parameter** | **r** | **95% CI** | ***p* Value** | **R²** | **r** | **95% CI** | ***p* Value** | **R²** | **r** | **95% CI** | ***p* Value** | **R²** |
| Total bilirubin | 0.00 | -0.32 – +0.24 | 0.732 | 0.03 | 0.33 | +0.05 – +0.52 | 0.011 | 0.11 | 0.21 | -0.02 – +0.42 | 0.064 | 0.01 |
| Lactate | 0.05 | -0.36 – +0.20 | 0.070 | 0.26 | -0.18 | -0.44 – +0.11 | 0.561 | 0.01 | -0.37 | -0.68 – +0.07 | 0.084 | 0.00 |
| LDH | 0.02 | -0.38 – +0.26 | 0.215 | 0.18 | 0.12 | -0.13 – +0.38 | 0.352 | 0.01 | 0.06 | -0.17 – +0.29 | 0.597 | 0.00 |
| P-Amylase | 0.05 | -0.58 – +0.34 | 0.100 | 0.25 | -0.01 | -0.26 – +0.27 | 0.949 | 0.00 | 0.14 | -0.30 – +0.53 | 0.536 | 0.00 |
| Lipase | 0.00 | -0.64 – +0.24 | 0.803 | 0.10 | 0.08 | -0.18 – +0.35 | 0.542 | 0.02 | 0.11 | -0.32 – +0.50 | 0.620 | 0.00 |
| AST | 0.03 | -0.37 – +0.25 | 0.161 | 0.17 | 0.20 | -0.04 – +0.46 | 0.133 | 0.01 | 0.32 | +0.09 – +0.51 | 0.005 | 0.11 |
| ALT | 0.02 | -0.33 – +0.30 | 0.262 | 0.14 | 0.09 | -0.02 – 0.48 | 0.480 | 0.00 | 0.27 | +0.05 – +0.48 | 0.017 | 0.05 |
| AP | 0.01 | -0.21 – +0.41 | 0.397 | 0.03 | -0.09 | -0.28 – +0.25 | 0.493 | 0.02 | 0.00 | -0.23 – + 0.24 | 0.974 | 0.01 |
| GGT | 0.02 | -0.36 – +0.26 | 0.238 | 0.11 | -0.18 | -0.42 – +0.09 | 0.171 | 0.04 | 0.02 | -0.21 – +0.26 | 0.842 | 0.00 |
| CRP | 0.00 | -0.34 – +0.27 | 0.970 | 0.02 | 0.07 | -0.23 – +0.32 | 0.595 | 0.00 | 0.29 | +0.04 – +0.50 | 0.019 | 0.00 |
| SCr | -0.25 | -0.52 – +0.04 | 0.085 | 0.05 | -0.03 | 0.31 – +0.21 | 0.674 | 0.00 | -0.20 | -0.41 – +0.03 | 0.076 | 0.03 |
| Urea | -0.19 | -0.46 – +0.11 | 0.208 | 0.07 | -0.02 | -0.28 – +0.23 | 0.852 | 0.01 | -0.01 | -0.24 – 0.22 | 0.939 | 0.00 |

Molecular Adsorbent Recirculating System (MARS); Therapeutic Plasma Exchange (TPE); Lactate Dehydrogenase (LDH); Pancreas-Amylase (P-Amylase); Aspartate transaminase (AST); Alanine transaminase (ALT); Alkaline phosphatase (AP); Gamma-glutamyl Transpeptidase (GGT); C-reactive Protein (CRP); Serum creatinine (SCr).

## Supplemental Figure

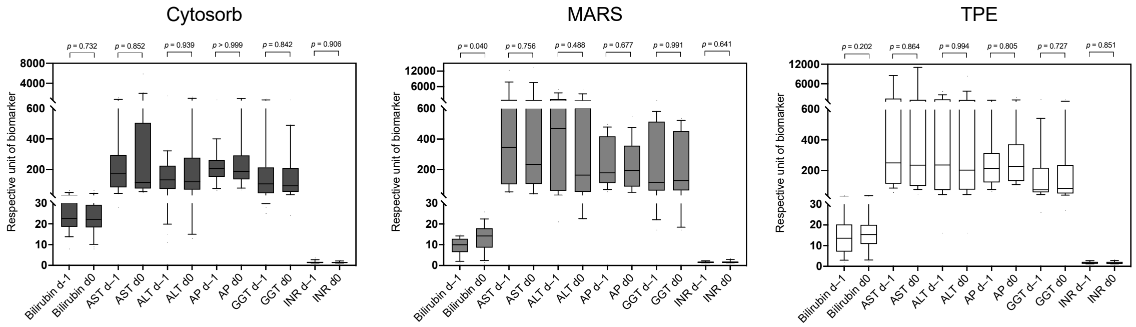


**Figure S1.** Course of routine liver parameters within two days before first liver support therapy.

Procedure-dependent absolute changes of blood pressure and vasopressor demand in response to Cytosorb, Molecular Adsorbent Recirculating System (MARS) and Therapeutic Plasma Exchange (TPE) therapy. Data are reported as box-and-whisker plots (interquartile range, 10% to 90% interval). D0 day of first liver support therapy, d–1 one day before first liver support therapy. Total bilirubin (Bilirubin) [mg/dL]; Aspartate transaminase (AST) [U/L]; Alanine transaminase (ALT) [U/L]; Alkaline phosphatase (AP) [U/L]; Gamma-glutamyl Transpeptidase (GGT) [U/L]; International Normalized Ratio (INR).
